# Intrahepatic mRNA Expression of FAS, FASL, and FOXP3 Genes Is Associated with the Pathophysiology of Chronic HCV Infection

**DOI:** 10.1371/journal.pone.0156604

**Published:** 2016-05-31

**Authors:** Ednelza da Silva Graça Amoras, Samara Tatielle Monteiro Gomes, Felipe Bonfim Freitas, Bárbara Brasil Santana, Geraldo Ishak, Marialva Tereza Ferreira de Araújo, Sâmia Demachki, Simone Regina Souza da Silva Conde, Marluísa de Oliveira Guimarães Ishak, Ricardo Ishak, Antonio Carlos Rosário Vallinoto

**Affiliations:** 1 Laboratory of Virology, Institute of Biological Sciences, Federal University of Pará (Universidade Federal do Pará –UFPA), Belém, Pará, Brazil; 2 João de Barros Barreto Hospital, Federal University of Pará (Universidade Federal do Pará –UFPA), Belém, Pará, Brazil; 3 School of Medicine, Institute of Health Sciences, Federal University of Pará (Universidade Federal do Pará –UFPA), Belém, Pará, Brazil; 4 Hepatology Outpatient Service, Holy House of Mercy Foundation of Pará (Santa Casa de Misericórdia do Pará), Belém, Pará, Brazil; University of Thessaly, Faculty of Medicine, GREECE

## Abstract

This study aimed to evaluate the relative mRNA expression of Fas receptor (*FAS*), Fas ligand (*FASL*), and forkhead box protein 3 (*FOXP3*) in liver biopsy specimens obtained from patients with viral and non-viral chronic hepatitis and correlate their expression with the fibrosis stage. A total of 51 liver biopsy specimens obtained from HBV (n = 6), HCV (n = 28), and non-viral hepatic disease (NVHD) (n = 9) patients and from individuals with normal liver histology (n = 8) (control—CT) were analyzed. Quantifications of the target genes were assessed using qPCR, and liver biopsies according to the METAVIR classification. The mRNA expression levels of *FAS* and *FASL* were lower in the CT group compared to the groups of patients. The increase in the mRNA expression of *FAS* and *FASL* was correlated with higher levels of inflammation and disease progression, followed by a decline in tissues with cirrhosis, and it was also associated with increased levels of alanine aminotransferase (ALT) and aspartate aminotransferase (AST). Higher mRNA expression of *FOXP3* was observed in the HCV and NVHD groups, with the peak observed among patients with cirrhosis. The increased *FOXP3* mRNA expression was positively correlated with increased *FAS* and *FASL* mRNA expression and the AST and ALT levels in all patients. **Conclusions:** These results suggest that regardless of the cause, the course of chronic liver disease may be modulated by the analyzed genes and correlated with an increase in regulatory T cells during the liver damage followed by hepatocyte destruction by Fas/FasL system and subsequent non specific lymphocytic infiltrate accumulation.

## Introduction

Injuries to the liver caused by the hepatitis B virus (HBV) and the hepatitis C virus (HCV) are mediated primarily by the host’s immune response to viral proteins expressed by infected hepatocytes and, to a lesser extent, by the direct cytopathic effects caused by the viruses [1]. Currently, viral hepatitis is a major pandemic, accounting for most forms of chronic liver disease found worldwide, and therefore, it is a relevant public health problem [2]. Approximately 170 million people, corresponding to 3% of the global population, with different patterns of geographic distribution, are living with chronic HCV, whereas approximately 7% of the global population is chronically infected with HBV [2].

The induction of apoptosis or programmed cell death, mediated by either extrinsic or intrinsic pathways, is one mechanism by which infected liver cells may limit the initial viral spread [3]. However, regardless of how it is initiated, it results in the activation of an extremely important and specific class of caspases that cleave cellular proteins and culminates in cell disruption [4]. The extrinsic pathway is triggered by signals that arise from the death receptors located on the cell surface. These receptors are activated by ligands, such as tumor necrosis factor-related apoptosis-inducing ligand (TRAIL) and FasL (CD95L), and the apoptosis is mediated by Fas and its ligand FasL, one of the most well-defined signaling pathways [3, 4]. The activation of caspase-8 via the Fas receptor is an important mechanism that initiates hepatocyte apoptosis in both physiological and pathological conditions, and it is extremely important in the pathophysiology of several liver diseases [4].

Under normal conditions, hepatocytes express low levels of the Fas receptor; however, the presence of inflammatory cytokines such as IL-1 or the presence of oxidative stress that results in DNA damage and *p53* activation can increase the expression of these receptors, making the cells more susceptible to apoptosis by the Fas system [1, 4]. Virus-infected cell apoptosis can be induced either by the host immune response through a mechanism involving cytotoxic T-cells and natural killer (NK) cells or by viral proteins. Apoptosis, by itself, has been considered a common method for interrupting viral replication and eliminating infected cells [1]. However, several viral genomes encode proteins that repress the process of apoptosis to escape the host immune response. Thus, these viruses are able to persist in the host’s body for years, contributing to the onset of chronic disease [1].

Regulatory T cells (Tregs) are a subpopulation of CD4^+^ T and constitutively express IL-2 receptor alpha-chain (CD25) on their surface. Tregs can block the effector function of CD4^**+**^CD8^**+**^ T cells, NK cells, and NKT cells, blocking the activation and function of these lymphocytes and therefore helping to maintain the homeostasis and peripheral tolerance to self-antigens. They perform their function by releasing inhibitory cytokines, such as IL-10 and TGF-β [5]. Despite the heterogeneity of the Treg cell population, except for TR1, all of them express the transcription factor forkhead box protein 3 (FoxP3), which is the major marker and functional regulator of Tregs [5].

The balance between effector T cells and regulatory T cells influences the resolution of various parasitic infectious diseases [6]. The role of CD4^+^CD25^+^FoxP3^+^ T cells in hepatitis B and C has been assessed, particularly in the chronic forms of the infection [6]. The elimination of HCV and HBV is associated with a vigorous virus-specific CD4^+^ and CD8^+^ T cell response during the acute phase of the infection. By contrast, viral persistence is associated with a poor and dysfunctional virus-specific T cell response [6]. There is strong evidence that different populations of Tregs mediate the suppression of virus-specific T cells in HCV and HBV infections. This immune suppression may not only contribute to the persistence of the virus but also protect against severe liver damage [6].

Regardless of the initial cause, continued liver damage causes inflammation, matrix deposition, parenchymal cell death, and angiogenesis, leading to progressive fibrosis. Furthermore, chronic liver infections caused by HBV and HCV or even non-viral liver diseases are important because these chronic carriers live with a potential risk of developing more severe complications, such as cirrhosis and hepatocellular carcinoma [2, 7].

Previous study from our group has reported the association between the *NGF* and *P75*^*NRT*^ gene expressions and the liver fibrosis stages due to viral and non-viral causes [8]. Into this perspective, the present work aims to continue to investigate the factors associated with fibrosis by quantifying expression of the *Fas*, *FasL*, and *FOXP3* genes in liver biopsy specimens obtained from patients with viral (HBV and HCV) and non-viral hepatic disease (NVHD) chronic hepatitis and correlate their possible roles in the pathogenesis and clinical presentation of these infections and in the various stages of fibrosis and hepatic inflammatory activity.

## Materials and Methods

### Study Population

The studied group consisted of 51 consecutive cases of untreated chronic carriers of HBV (n = 6), HCV (n = 28), and NVHD (n = 9) (including non-alcoholic liver disease, autoimmune hepatitis, and primary biliary cirrhosis, among others) attend at the Hepatology Outpatient Service of the Santa Casa de Misericórdia do Pará Foundation Hospital (Fundação Santa Casa de Misericórdia do Pará—FSCMPA). The control group (CT) was composed by eight (n = 8) selected patients undergoing conventional cholecystectomy without hepatic necroinflammatory changes at the Surgery Service of João de Barros Barreto University Hospital (Hospital Universitário João de Barros Barreto) at the Federal University of Pará (Universidade Federal do Pará—UFPA). Further details about the criterias for patients selection can be found in our previous report [8].

### Ethics

The present study was submitted and approved by the Santa Casa de Misericórdia do Pará Foundation Hospital Research Ethics Committee (protocol No. 117/2009 and 684.432/2014) and followed the Guidelines and Rules for Research Involving Human Subjects (Resolutions 196 and 240 of the National Council of Health). All subjects who agreed to participate in the study signed a Free and Informed Consent Form (FICF).

### Sample Collection

As described previously [8] liver biopsy specimens were obtained from patients who were referred for further investigation of hepatic parenchymal alterations. Ultrasound-guided Tru-Cut liver biopsies were performed and the diagnosis followed the French METAVIR scoring system [9]. Part of the biopsy specimens was sent for genetic studies at the Laboratory of Virology/ICB/UFPA and stored at -70°C until testing. Blood samples were also collected in vacuum tubes containing EDTA as an anticoagulant and plasma was separated by centrifugation and stored at -20°C until use for biochemical and viral markers measurement [8].

### RNA Extraction and Reverse Transcription (cDNA)

Liver tissue fragments were kept in 500 μL of *RNA later® Tissue Collection* (Ambion, ThermoFisher Scientific Inc., Waltham, MA USA) solution for RNA preservation. RNA was subsequently extracted and transcribed into complementary DNA (cDNA) as previously described [8].

### mRNA Quantification by Real Time PCR (qPCR)

Real-time PCR (qPCR) was performed in 96-well plates using TaqMan^TM^ reagents (Applied Biosystems, USA) on a Step One Plus machine (Life Technologies, Carlsbad, CA, USA). For both the patient groups and the control group, expression assays for the *FAS* (Fas Hs00163653_m1), *FASL* (FasL Hs00181225_m1), *FOXP3* (FOXP3 Hs01085834_m1) and glyceraldehyde-3-phosphate dehydrogenase (*GAPDH*) genes (P/N 4326317E, Life Technologies, CA, USA) were performed in separate wells (singleplex) with commercial primers obtained from Life Technologies (Carlsbad, CA, USA). The relative expression of each gene was performed as described by Amoras et al. [8].

### Statistical Procedures

As reported by Amoras et al. [8], all mRNA expression data are presented as medians, and serum dosages are presented as means. The statistical analysis was performed using the GraphPad Prism 5.0 [10] and BioEstat 5.0 [11] softwares and, as appropriate, Kruskal-Wallis test and the Mann-Whitney U-test were used for analyzing the differences between the groups. The relationships between two variables were determined using Spearman correlation analysis. The significance level was set at 5% (p ≤ 0.05).

## Results

### Biochemical and Histopathological Analysis

Patients enrolled in the present study were previously investigated in relation to their biochemical profile (ALT, AST, GGT levels) as well as the stages of fibrosis and inflammation stages as described, in details, by our group [8]. Additionally, in the present analysis, we observed that the inflammation process was absent in 50% of patients classified as stages F0 of liver fibrosis and in 23.53% of patients classified as stages F1. For all other scores, mild (A1) and moderate (A2) levels of inflammation were observed; a higher frequency (71.43%) of stage A2 was observed among individuals classified as stage F2 (Table 1). No case of A3 level of inflammation was reported among the patients.

**Table 1:** Frequency of inflammatory activity levels among the liver fibrosis scores of with viral hepatitis and non-viral hepatic disease.

Inflammatory activity levels^b^	Liver fibrosis scores^a^ (n) %				
	F0	F1	F2	F3	F4
**A0**	(2) 50	(4) 23.53	0	0	0
**A1**	(2) 50	(9) 52.94	(2) 28.57	(2) 50	(4) 50
**A2**	0	(4) 23.53	(5) 71.43	(2) 50	(4) 50

^a^ Fibrosis scores F0: no fibrosis; F1: portal fibrosis without septa; F2: portal fibrosis with few septa; F3: numerous septa without cirrhosis; F4: cirrhosis

^b^Inflammatory Activity A0: absent; A1: minimum; A2: moderate.

**Fig 1:**
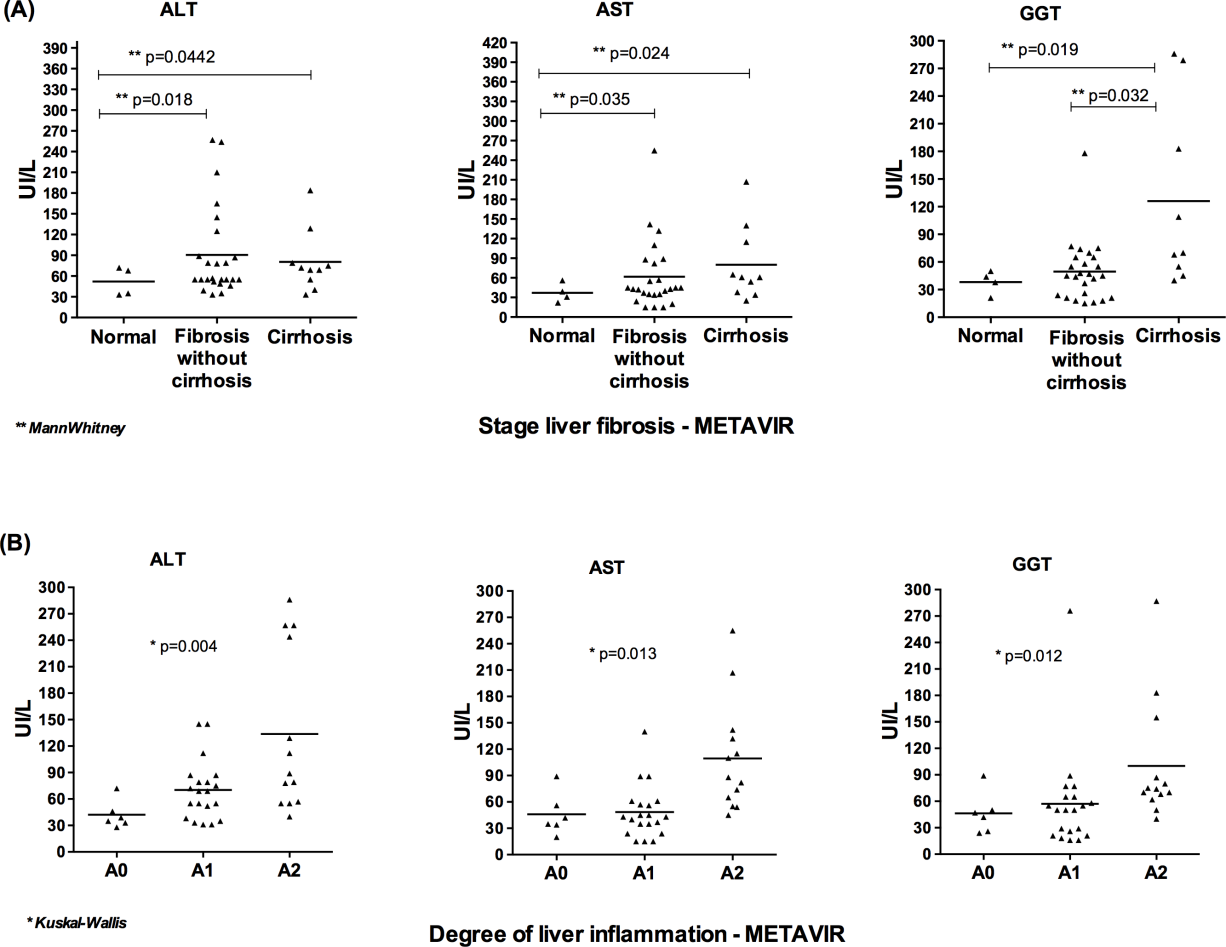
Mean plasma ALT, AST and GGT levels of all patients with viral and non-viral chronic hepatic disease. A: patients without histological changes in the liver, with fibrosis (without cirrhosis) and with cirrhosis. B: according to the inflammatory activity.

Fig 1A shows the analysis of the mean levels of liver enzymes among patients without histological changes in the liver and in those with advanced fibrosis and cirrhosis. This analysis reveals that the ALT and AST levels were significantly higher among the group of individuals with fibrosis and cirrhosis than among the group of individuals without histological changes in the liver (p = 0.018 and p = 0.0442, respectively). GGT levels were significantly higher in the group of individuals with cirrhosis compared to individuals from the normal and fibrosis groups (p = 0.057 and p = 0.022, respectively). Regarding the inflammatory process, serum ALT, AST, and GGT levels were significantly higher in the group of individuals with the inflammatory activity of A2 compared to individuals classified as A0 and A1 (mild liver inflammation) (Fig 1B).

### mRNA Levels of the *Fas*, *Fasl* and *Foxp3* in Liver Tissue

The mRNA expression levels of the genes studied were measured in the groups of patients with chronic liver disease and compared with the results obtained in the control group, considering the values expressed in fold change relative to the reference calibrator.

**Fig 2:**
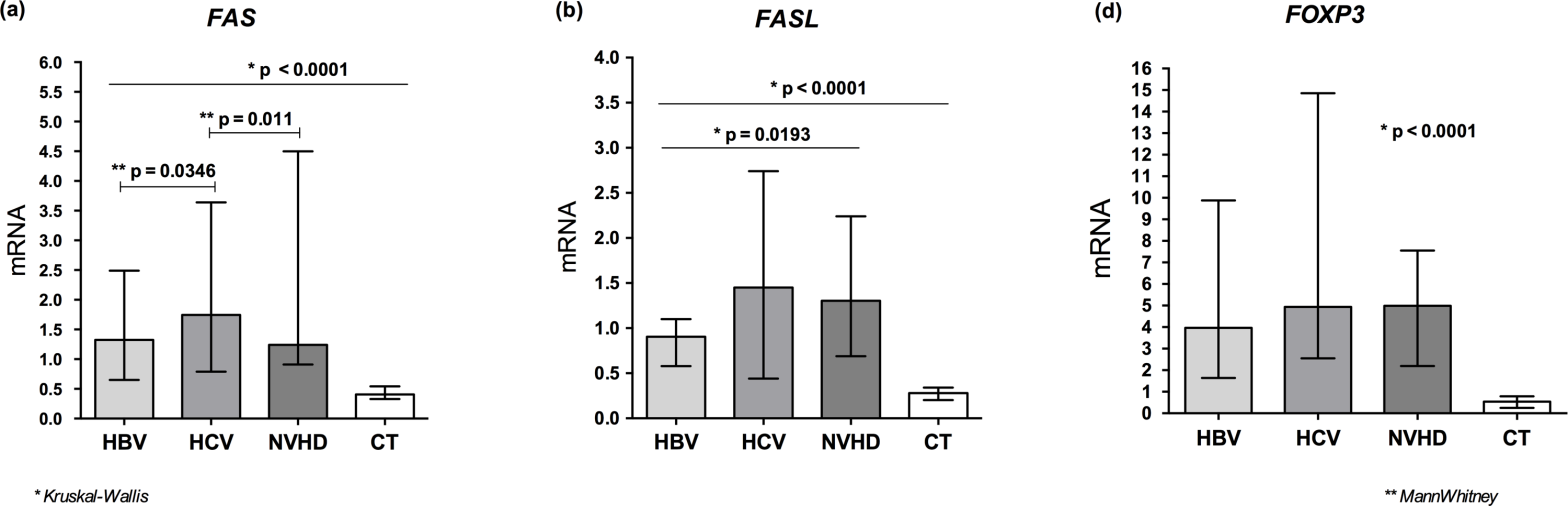
Relative quantification mRNA levels *FAS*, *FASL and FOXP3* in liver tissue. A: mRNA levels *FAS* receptor in the groups of patients with HBV, HCV, and NVHD and the control group. B: mRNA levels *FASL* ligand in the groups of patients with HBV, HCV, and NVHD and the control group. C: mRNA levels *FOXP3* in the groups of patients with HBV, HCV, and NVHD and the control group.

The mRNA expression level of the *FAS* receptor and *Fas* ligand (*FASL*) was higher in patients with HCV, whereas in groups with HBV and NVHD, gene expression levels were lower. Significant differences were observed when these groups were compared to the control group (p < 0.0001) (Fig 2). The expression of the *FAS* receptor was significantly different between the groups: HBV and HCV (p = 0.034) and HCV and NVHD (p = 0.011) (Fig 2A); the expression of *FASL* was significantly different between all groups (p = 0.0193) (Fig 2B). The mRNA levels of the transcription factor *FOXP3* were significantly higher in the patients than in the control group (p < 0.0001); among the patients, the HBV group presented lower mRNA expression levels compared to the HCV and NVHD groups, which presented the highest gene expression levels; however, these differences were not significant (Fig 2C).

### Relative Quantification mRNA Levels of the *Fas*, *Fasl* and *Foxp3* Genes in the Liver Tissue

**Fig 3:**
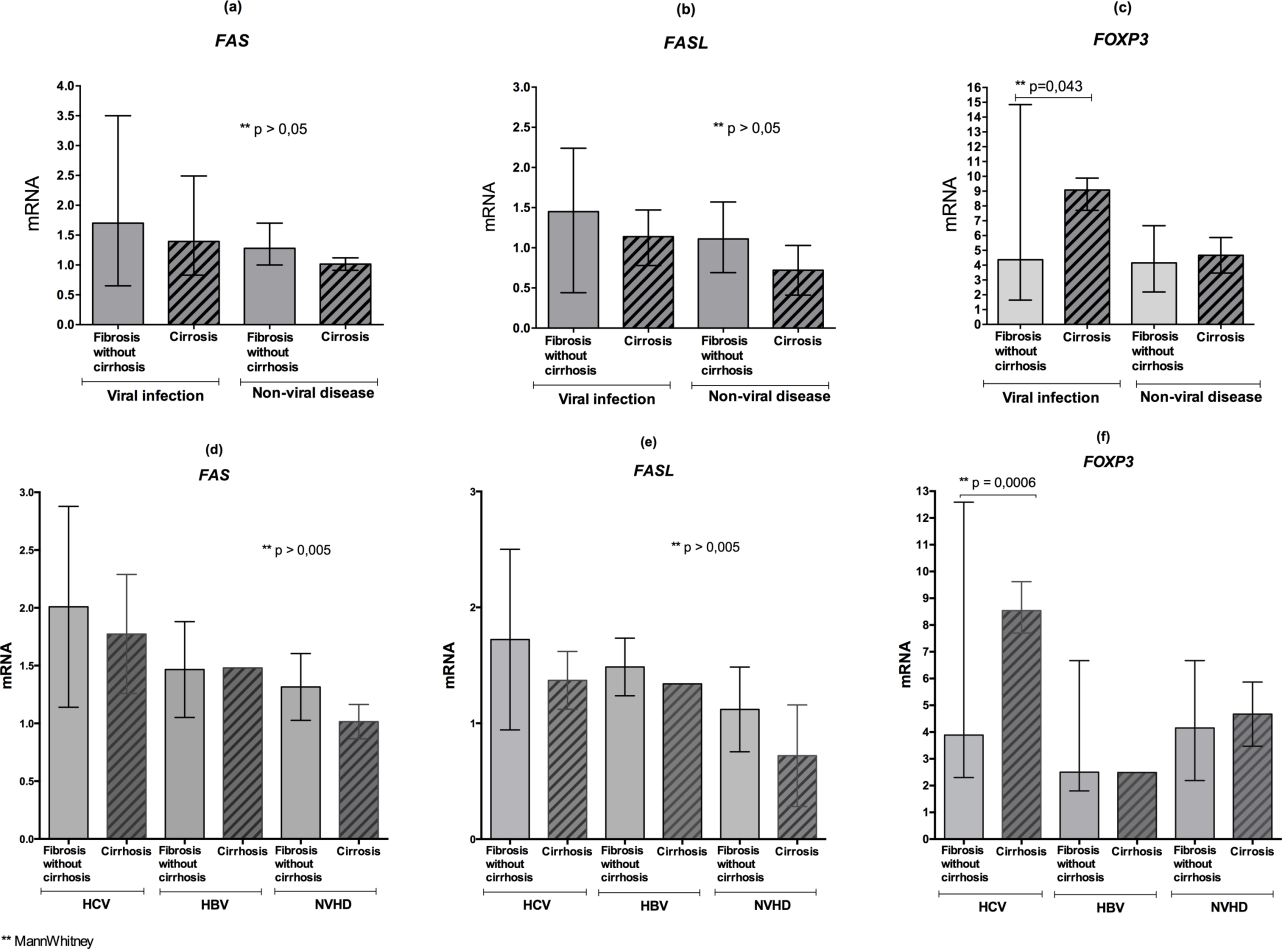
mRNA levels of *FAS*, *FASL* and *FOXP3* in the groups without cirrhosis and with cirrhosis. A-C: Quantification of the *FAS* receptor, *FASL* ligand and *FOXP3* mRNA levels in the groups with fibrosis (without cirrhosis) and the groups with cirrhosis with viral and non-viral liver disease. D-F: Quantification of the *FAS* receptor, *FASL* ligand and *FOXP3* mRNA levels in the groups with hepatic fibrosis (without cirrhosis) and cirrhosis due to viral and non-viral causes.

Fig 3 shows that, according to the clinical presentation, considering the type of infection and the chronic liver disease, the fibrosis group (without cirrhosis) presented the highest expression of *FAS* (Fig 3A) and *FASL* (Fig 3B) compared to patients with cirrhosis (both viral and non-viral); however, this difference was not significant. By contrast, only among the group of patients with viral infection, the expression of the transcription factor *FOXP3* was significantly higher (p = 0.043) in cirrhotic patients compared to those with only fibrosis (Fig 3C); this finding was not observed among patients with non-viral hepatic disease.

When analyzing this group in particular, it was observed the same profile of high expression of *FAS* and *FASL* in the group with HCV, followed by groups with HBV and NVHD, but there was no statistical significance (Fig 3D and 3E). The expression of *FOXP3* was significantly higher in patients with HCV cirrhosis (p = 0.0006) with the same result observed in the group with NVHD, although not statistically significant. In the group with HBV, this difference was not observed (Fig 3F).

### mRNA Levels of *FAS*, *FASL* and *FOXP3* in Liver Tissue According to the Fibrosis Stages and Inflammatory Activity

**Fig 4:**
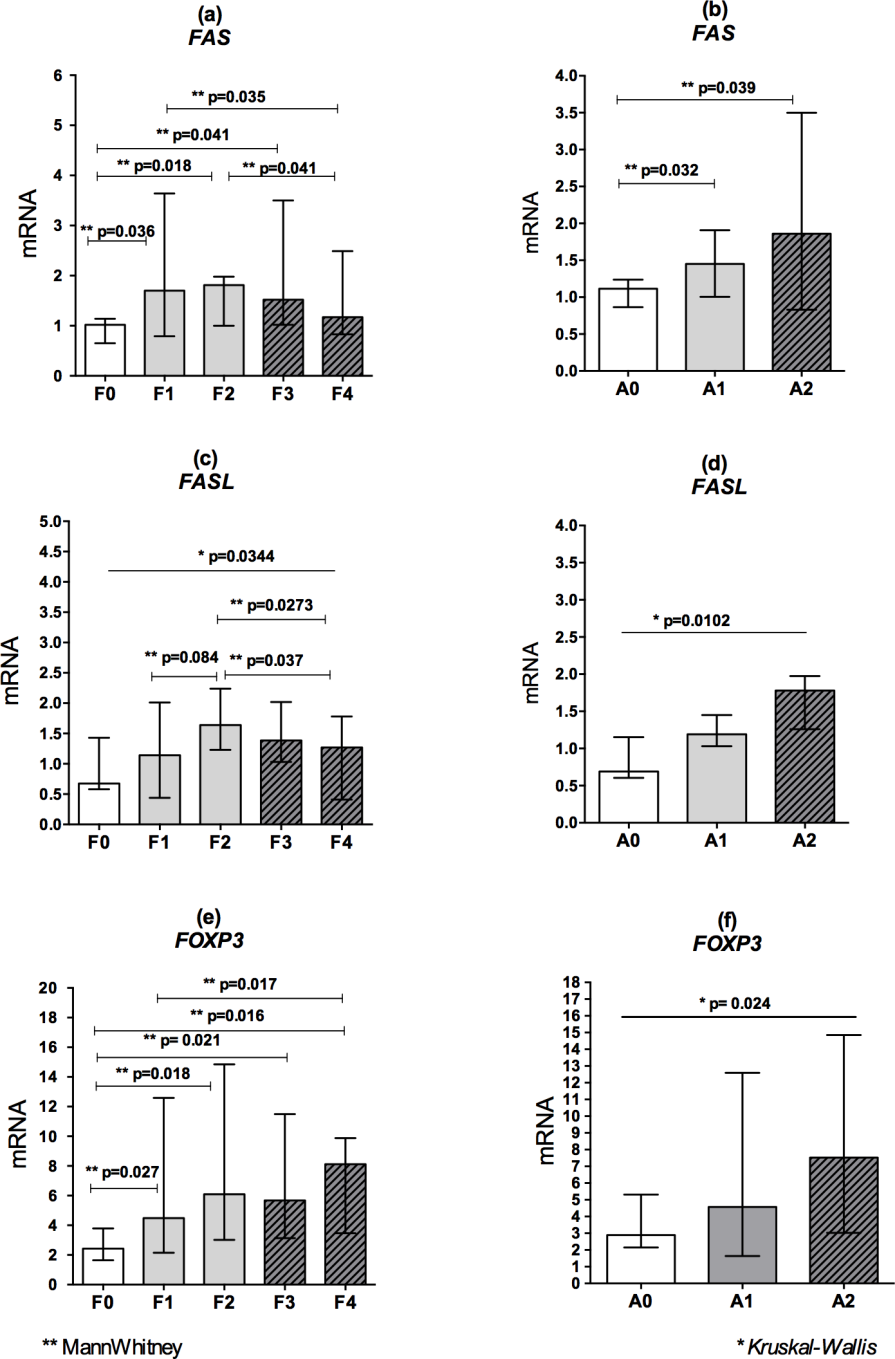
mRNA levels of *FAS*, *FASL* and *FOXP3* according to the clinical conditions of liver. A-F: mRNA levels of *FAS*, *FASL* and *FOXP3* according to the fibrosis stages (F0 to F4) and inflammatory activity (A0 to A2) in the liver tissue of patients with viral and non-viral chronic liver disease (METAVIR).

Fig 4 shows that, when all chronic patients were grouped, the mRNA levels of *FAS* (Fig 4A) and *FASL* (Fig 4C) were lower in patients classified as stage F0 and significantly increased towards stages F1 to F2, followed by a decline in F3 and F4 (p = 0.035 and p = 0.041, respectively). The mRNA levels of *FASL* (Fig 4C) were significantly higher in patients classified as stage F2 compared to other fibrosis scores (p < 0.05). Regarding the liver inflammation process, the mRNA levels of *FAS* (Fig 4B) and *FASL* (Fig 4D) were lower in patients classified as stage A0 and significantly increased in stages A1 and A2.

The mRNA levels of the transcription factor *FOXP3* were lower in groups of patients without fibrosis (stage F0) (Fig 4E) and significantly increased in other stages of liver injury (p < 0.05). The same finding was observed for liver inflammatory activity (Fig 4F), in which the mRNA levels of this transcription factor were significantly higher with increasing levels of inflammation (p = 0.024).

**Fig 5:**
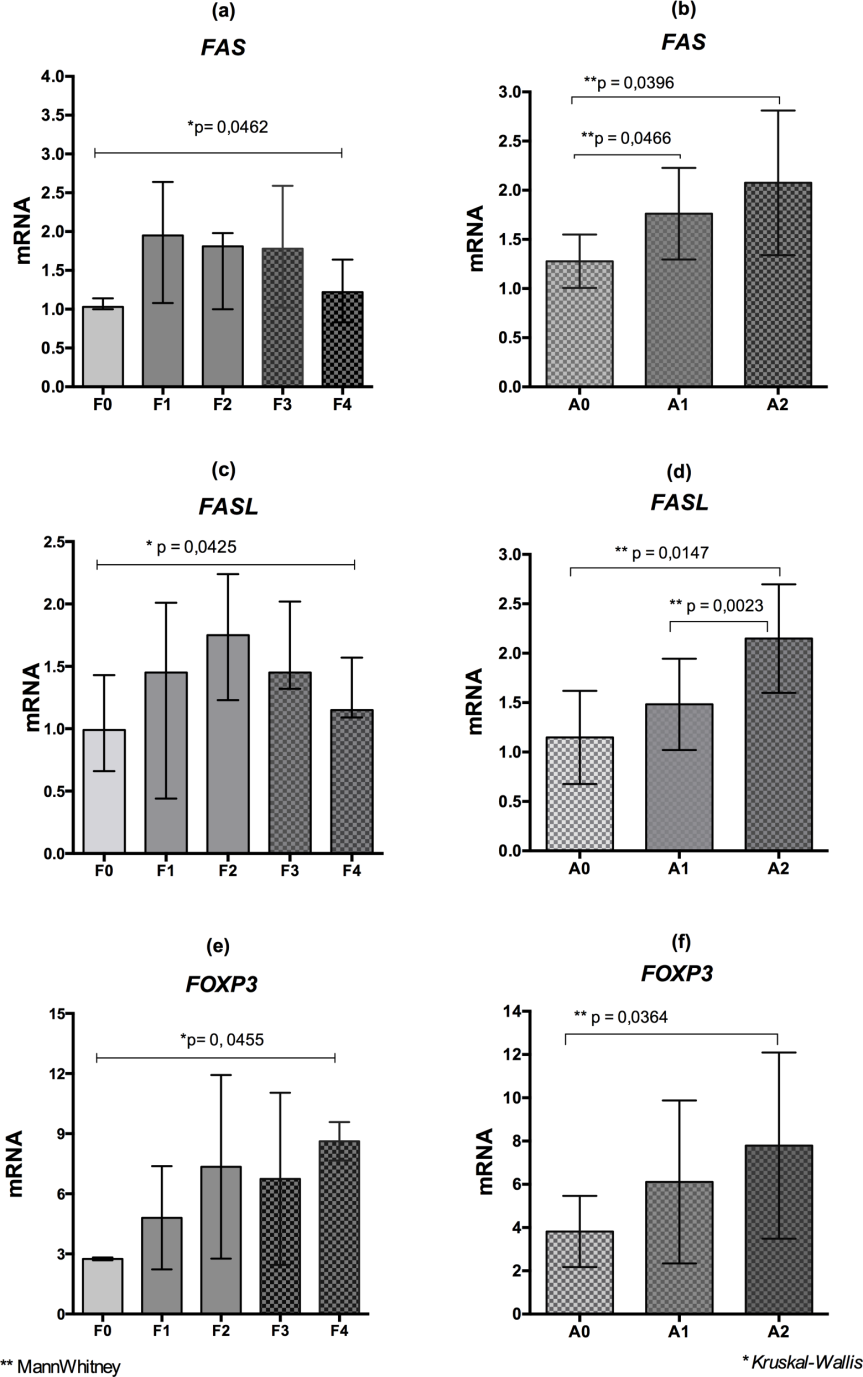
mRNA levels of *FAS*, *FASL* and *FOXP3* according to the clinical conditions of liver in the HCV infection. A-F: mRNA levels of *FAS*, *FASL* and *FOXP3* according to the fibrosis stages (F0 to F4) and inflammatory activity (A0 to A2) in the liver tissue of patients with HCV (METAVIR).

All of the liver fibrosis scores (METAVIR) was observed in the groups with HCV (Fig 5) and FAS and FASL expression levels were also lower in the initial phase (F0) and the final stage (F4) of the disease, with statistical significance considering the intermediate stages (p = 0,0462; Fig 5A and 5B); it is important to notice that there was an increase of the gene expressions significantly associated with higher levels of inflammation (Fig 5B and 5D).

The increased *FOXP3* gene expression was significantly associated with progression of liver disease (p = 0.0455; Fig 5E) and with the mild (A0) and severe (A2) inflammatory activity (p = 0,0368; Fig 5F).

The group with HBV (n = 6) showed only F0, F1 and F4 scores, while in the NVHD (n = 8) group there was no F2 score, impairing the analysis of gene expression in accordance with the progression of chronic liver disease.

### Correlation of *FAS* and *FASL* with *FOXP3* mRNA Levels in Liver Tissue

**Fig 6:**
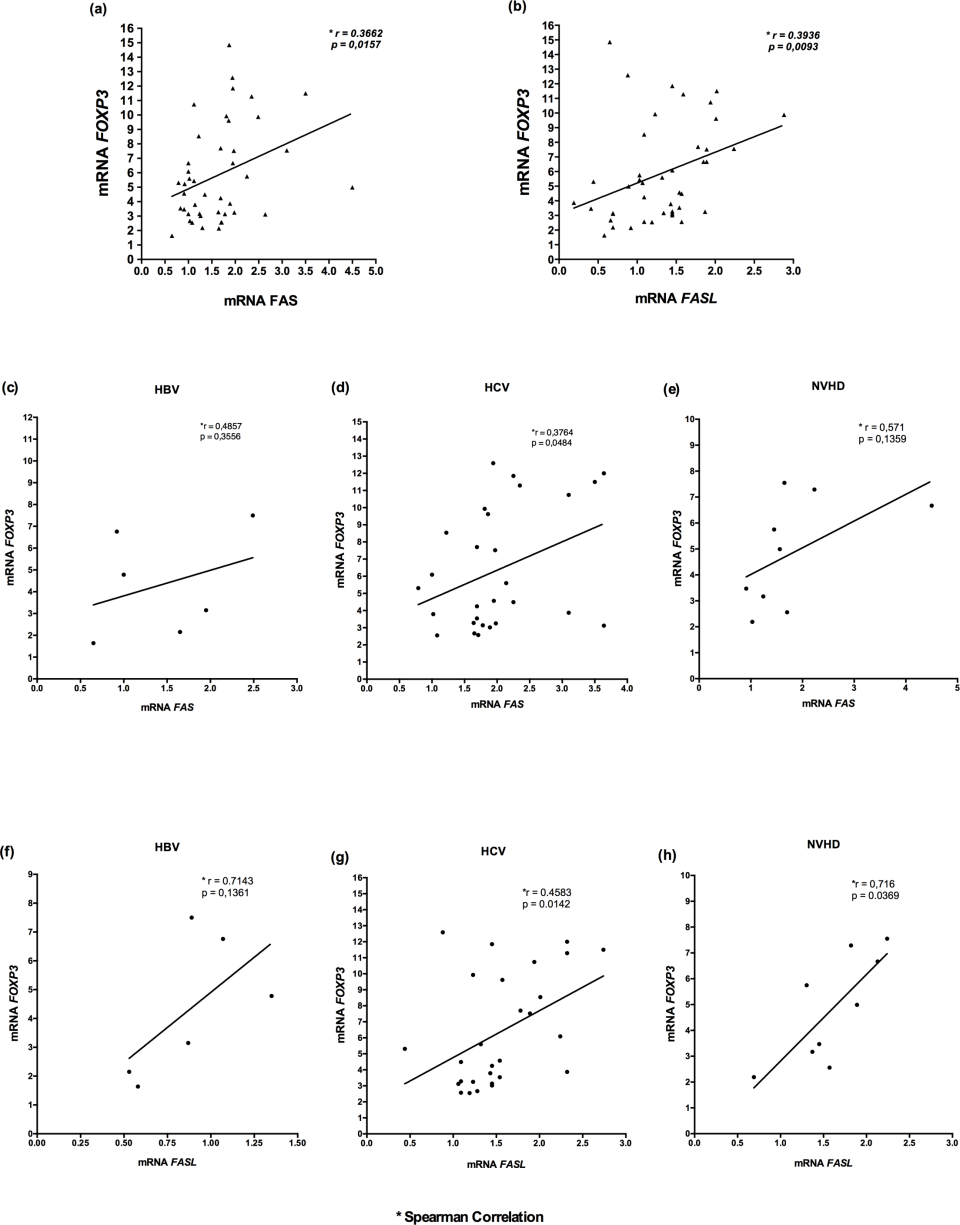
Correlation of *FAS* and *FASL* with *FOXP3* mRNA levels in liver tissue. A-E: Spearman correlation analysis with mRNA levels between *FOXP3* and the *FAS* receptor and between *FOXP3* and the *FASL* ligand in the chronic liver disease group (A and B), and according to the groups HBV (C and F), HCV infections (D and G), and NVHD (E and H).

In the group of patients herein studied, there was a significant positive correlation between the *FOXP3* mRNA levels and *FAS* receptor (p = 0.0157) (Fig 6A) and *FASL* (p = 0.0093) (Fig 6B). The observation of the groups separately, shows that this correlation profile was present in all groups. However, the significance of correlation between *FAS* and *FASL* with *FOXP3* was observed solely in the group with HCV (p = 0.0484 and p = 0.0142; Fig 6D and 6G). The correlation between *FASL* and *FOXP3* was also significant in the group with NVHD (p = 0.0369; Fig 6H).

### Correlation of *FAS* and *FASL* with *FOXP3* mRNA Levels in Liver Tissue According with ALT and AST

**Fig 7:**
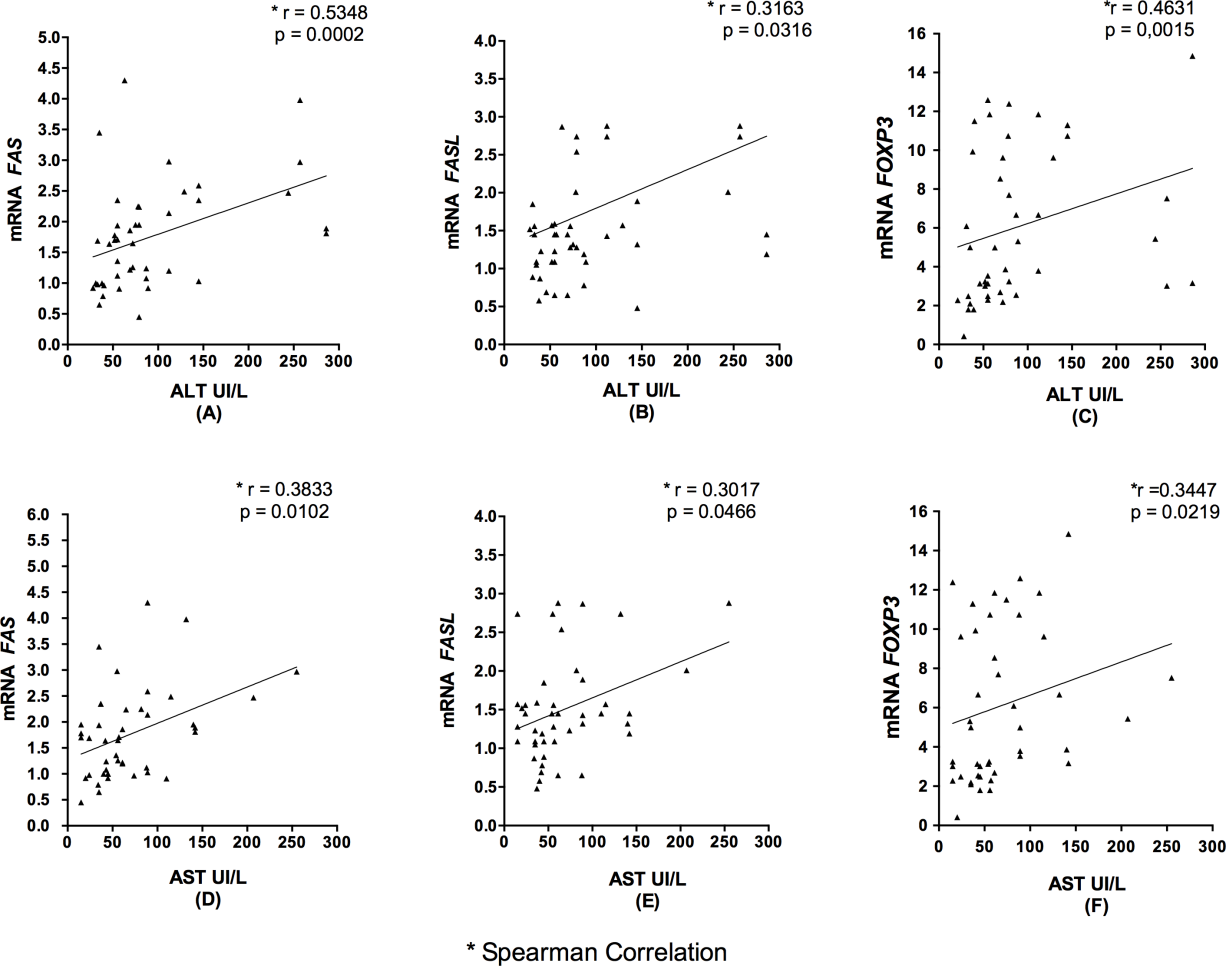
Correlation of *FAS*, *FASL* and *FOXP3* mRNA levels with ALT and AST concentrations. A, D: Spearman correlation analysis between the *FAS* receptor mRNA levels and plasma ALT and AST concentrations. B, E: Spearman correlation analysis between the *FASL* mRNA levels and plasma ALT and AST concentrations. C, F: Spearman correlation analysis between the *FOXP3* mRNA levels and plasma ALT and AST concentrations, in the chronic liver disease group.

Fig 7 shows that, in the groups of hepatitis analyzed, there was a positive correlation of mRNA levels of *FAS* (Fig 6A and 6D) (p = 0.0002 and p = 0.0136), *FASL* (Fig 6B and 6E) (p = 0.0102 and p = 0.0466), and *FOXP3* (Fig 6C and 6F) (p = 0.0015 and p = 0.0219) with the liver enzymes ALT and AST; these associations were found to be significant.

## Discussion

In the present study, there was a clear predominance of mild and moderate fibrosis stages and inflammatory activity (A0, A1 and A2) with mean ALT levels higher than AST, a characteristic pattern of untreated infections and chronic liver disease at early and intermediate stages [12, 13]. All patients with HBV were HBeAg negative and presented the lowest mean levels of ALT and AST. This finding is in line with that of a previous study showing a reduction in ALT activity levels during seroconversion of HBeAg-positive patients, which suggests that ALT is useful not only in determining the presence of hepatitis B and the need for treatment but also in measuring the natural course of the infection and predicting HBeAg seroconversion [14].

Higher scores of fibrosis and liver inflammation were observed in the group of patients with HCV and NVH and corresponded to the highest intrahepatic mRNA expression of *FAS*, *FASL*, and *FOXP3*. These findings support the hypothesis that hepatocyte apoptosis is significantly involved in HCV pathogenesis [15, 16], involving at least 3 regulatory mechanisms: (i) the HCV core protein, leading to suppression [17] or induction of apoptosis [18]; (ii) 2 HCV envelope proteins E1 [19] and E2 [20], through the induction of cell death; and (iii) HCV non-structural (NS) proteins that exert an anti-apoptotic effect [21, 22]. This apoptosis regulation would be beneficial for the virus because it would prevent premature hepatocyte death before complete virus replication and assembly processes [21].

The highest level of *FAS*, *FASL*, and *FOXP3* mRNA observed among patients with HBV, HCV, and NVH compared to the control group, which emphasizes the importance of these molecules in the pathophysiology of the immune response in situations of both viral and non-viral liver damage. Under normal conditions, hepatocytes express low levels of the Fas receptor; but the presence of inflammatory milieu or the presence of oxidative stress that results in DNA damage and *p53* activation can increase the expression of these receptors, making the cells more susceptible to apoptosis by the Fas system [1, 4]. Moreover, high levels of *FOXP3* mRNA suggests that the intrahepatic Tregs are involved in the regulation of chronic liver disease, most likely because they constitute an important part of lymphocyte infiltration into the portal space and hepatic lobules [22–25].

The observation of higher levels of expression of apoptotic genes and FOXP3 in HCV infected patients agrees with previous studies that reported cellular immune responses playing an important role in the immunopathogenesis of HCV infection, since viral clearance is associated with vigorous and multispecific HCV-specific T cell responses during acute infection [6]. In contrast, decreased production of cytokines and cytotoxic functions of the virus-specific CD8 + T cells are a feature of chronic HCV infection and contribute significantly to the persistence of viral infection [6]. Several mechanisms responsible for these disorders, in chronically infected patients, have been proposed, including HCV variants with altered epitope sequences, induction of anergy by high levels of antigen, impaired production of interferon gamma (IFN-γ), and the lack of various auxiliary functions or suppressive activity of regulatory T cells [6, 26].

By contrast, the lower expression of *FAS* and *FASL* mRNA observed in the group of patients with HBV (HBeAg^-^) compared to the HCV group indirectly corroborates the studies that demonstrate the induction of apoptosis in HBV-infected hepatocytes (HBeAg^+^) as a powerful antiviral defense mechanism that interrupts the spread of the virus [27], supporting the importance of the Fas/FasL interactions not only in the generation of damage in the infected hepatocytes but also potentially in the induction of apoptosis in cytotoxic T lymphocytes, which leads to viral persistence [28].

The lower expression of *FOXP3* mRNA in the HBV group (HBeAg^-^) observed in the present study may be explained by data from previous studies [29, 30] that demonstrate a positive correlation between the high HBV-DNA load and the high concentration of Treg in the liver, without controlling viral replication [29], in addition to a down regulation of *FOXP3* expression in patients with remitted disease compared to those diagnosed with active disease and without any treatment [30]. Germanidis et al. [29] reported down-regulated liver mRNA expression of FoxP3 and suppressive cytokines, IL-10, and TGF-β, in patients maintained on-treatment following 5 years of remission compared to patients with active disease and no prior treatment. CD8 was also decreased in patients on-treatment and in remission. This data suggest a decrease in both intra-hepatic FoxP3+ Tregs and CTLs following CHB resolution. Interestingly, IL-2 and IFN-γ expressions were not restored during remission; this could indicate long-term CTL impairment or else it could be due to a reduction in intra-hepatic CTLs preventing any immune response from being restored to pre-infection intensity. Our results of *FOXP3* mRNA expression can be explained also by the fact that during inflammatory expansion, HBV-specific Tregs might also be generated in response to HBsAg on infected hepatocytes explaining differing reports regarding HBV-specific and non-specific Tregs from chronic HBV patients [31].

Given that the levels of *FAS*, *FASL*, and *FOXP3* expression in the phases of chronic liver disease were not significantly different between viral infection and non-viral disease, all study subjects were grouped into a single group in an attempt to investigate the possible relationships between the expression of these genes and histological changes in the liver. In line with other studies [30–34], the present study demonstrated an increase in the expression of *FAS* and *FASL* mRNA in the progression of liver disease (F1 to F3) followed by a decline in the cirrhosis condition (F4). These findings indicate a modulation of apoptosis pathways during the course of chronic liver disease and demonstrate that apoptosis is inhibited as the disease progresses, leading to the immortalization of activated hepatic stellate cells and the development of cirrhosis. In addition, an increasing possibility of liver carcinogenesis is observed, especially with the increased proliferation rate and acquisition of genetic damage. These data demonstrated that the extrinsic apoptotic pathway (Fas-FasL) plays a direct and significant role in liver cell damage. It should be noted that, in the present study, the expression of *FASL* was significantly higher in the F2 fibrosis stage (moderate), in which the highest level of inflammation (A2) was also more frequent. This finding can be explained by the fact that the *FASL* gene causes pro-inflammatory activities by stimulating the secretion of IL-1β, which is responsible for the infiltration of neutrophils, justifying the presence of severe inflammation at this stage of the disease [34, 35]. Previous studies have shown that increased expression of the *FASL* gene induces apoptosis of T lymphocytes, which facilitates viral persistence and indirectly increases the likelihood of progression to cirrhosis and hepatocellular carcinoma [35–37].

The present study demonstrated that a substantial increase in *FAS*, *FASL*, and *FOXP3* mRNA expression was associated with the intensity of inflammation and serum AST and ALT levels. In addition, a positive correlation was observed between *FAS* and *FASL* expression and *FOXP3* expression, suggesting that the Fas/FasL system may be essential in chronic liver disease because elevated transaminase levels reflect the destruction of a large number of hepatocytes in chronic active hepatitis [38] and, as the inflammation is exacerbated and the advanced fibrosis (cirrhosis) is established, the mRNA expression of these apoptosis mediators decreases. This finding can be attributed to the evolution of liver damage, followed by the destruction of hepatocytes and the accumulation of lymphocytic infiltrate, including Tregs, suggesting persistent liver inflammation regardless of cause. This phenomenon seems to represent a major factor that contributes to the expansion of local Tregs [25]. Supporting this hypothesis, an association between higher levels of ALT and fibrosis was observed in the present study, whereas AST was higher among patients with cirrhosis. This finding suggests that the degree of fibrosis affects the amounts of these enzymes because AST levels that exceed ALT levels indicate an additional release of AST from the mitochondria of hepatocytes as a result of more severe or prolonged hepatocellular damage [39, 40]. It has been proposed that FoxP3+ Treg cells might be expanded, non-specifically, in response to chronic liver inflammation rather than as HBV-specific Tregs [30]. This study also reported a positive correlation of PD-1, PD-L1, and the apoptotic mediators FAS and FAS-L with inflammation intensity [30]. Current data confirm that FoxP3 is strongly correlated with inflammation in chronic HBV, in addition to PD-1, PD-L1, and CD8. FoxP3 expression also correlated closely with serum viral load, ALT, and AST [31].

However, the findings from studies that address the association between these apoptotic genes and inflammation and ALT levels in patients infected with HCV are contradictory [41, 42]. The positive association between the degree of fibrosis and inflammatory activity with increased GGT levels supports studies that explained the higher levels of GGT due to lesions in the bile ducts associated with AST levels and the METAVIR score in patients with HCV [41, 42].

Considering Treg cells would inhibit the activity of the effector T cells and NK cells inducing the apoptosis of target cells via death receptor signaling pathways, the increased Treg cells should inhibit apoptosis induced by cytotoxic immune cells. Probably, the association of FOXP3 expression with apoptotic genes and inflammation are justified not only by viral persistence and action of the viral proteins in the regulation of the expression of apoptotic genes [17, 18, 20, 21], but also corroborates the study by Morgan et al. [43] who utilized several CD4^+^ T cell clones to determine that CD25^+^ population within this cell line were able to proliferate and secrete IFN-γ to the same extent as the CD25^−^ population [43]. Therefore, it is likely that FOXP3 expression observed during HCV infection, confers regulatory activity predominantly, though the CD4^+^CD25^+^ expression may be responsible for the maintenance of effector activities in this context. Interestingly, Speletas et al. [25], also observed a high intrahepatic expression of another death ligand activating the caspase cascade and apoptosis, namely, TRAIL followed the same pattern of expression of Fas, the early stages of liver inflammation. As such, the elevated FasL expression, mainly expressed by CTLs, can also be explained, despite the fact that it is not followed by a parallel increase of TRAIL expression [25].

The results of the present study partially agree with previous studies in which the authors demonstrated, by immunohistochemistry and cell culture, that, in patients with HCV, the mean Foxp3^+^ Tregs are strongly correlated with liver inflammation scores, showing an increase in fibrosis and a decrease in cirrhosis. These findings suggest that, during the early stages of the disease, Foxp3^+^ Tregs modulate the effector functions of CD4^+^ and CD8^+^ T cells and, during the final phase (cirrhosis), when the environment and the liver architecture are changed, Tregs would already have been depleted due to excessive effector T cells [22, 44]. Different results show that, in chronic HCV infection, Foxp3^+^ Tregs of hepatic infiltration may limit fibrosis with a key role in suppressing the excessive immune activation induced by HCV because the ALT levels and HCV viral load do not correlate with the number of Foxp3^+^ Tregs in the liver [44, 45].

The discrepancy between the results of the present study and those of previous studies may be explained by the different methodologies used. Previous studies employed immunohistochemistry and flow cytometry in lymphocytic infiltration, whereas in the present study, *FOXP3* mRNA was quantified in liver biopsies by real-time PCR (qPCR). Therefore, gene expression was measured not only in the lymphocytic infiltration in the liver but also in all cell structures that favor the expression of the transcription factor *FOXP3*. It is known that, in chronic hepatitis, one of the mechanisms by which apoptosis promotes inflammation is associated with the activation of the Kupffer cells, which are the resident macrophages in the liver [46]. After the phagocytosis of apoptotic cells, Kupffer cells express death ligands, including FasL, which can induce hepatocyte apoptosis. This event may further aggravate liver inflammation, which can lead the hepatic stellate cells (HSC) to undergo a process of activation, TGF-β production, and transformation into a myofibroblast phenotype, promoting the development of fibrosis [46].

It is unclear how Tregs are correlated with liver fibrosis and inflammation. One possibility is that IL-10 produced by Tregs inhibits the deposition of the collagen matrix by hepatic stellate cells, decreasing fibrosis [47]. Alternatively, the secretion of TGF-β, also produced by Tregs, is an important factor for the local survival of Tregs and their function [48, 49]. However, TGF-β activates the hepatic stellate cells, decreasing the regeneration of hepatocytes, which promotes fibrosis [46]. In addition, other liver cells, including the sinusoidal epithelial cells [50] and Kupffer cells [51], produce TGF-β constitutively, indicating that TGF-β produced from other cells can act with the hepatic stellate cells to induce Foxp3^+^ Tregs [47, 52]. These findings can also explain the highest expression of *FOXP3* mRNA among patients with cirrhosis observed in the present study, with 50% of patients with cirrhosis having elevated levels of inflammatory activity (A2), which may be attributable to the most recent collagen formation in this group, characterized by the histopathological profile, as demonstrated in a previous study by our group [53].

The present results demonstrate that normal liver contains a low frequency of regulatory T cells, however, autoimmune and inflammatory diseases of the liver are associated with the enrichment of subsets of effector and regulatory T lymphocytes, often determining the outcome of hepatitis [54]. If the initial tissue injury is removed, followed by the regeneration of hepatocytes and biliary epithelial cells, it can lead to full recovery of liver tissue. However, with the persistence of injury (due to infection with HBV and HCV, or due to an autoimmune disease), acute hepatitis caused by effector T cells, is not controlled by the regulation of the immune system, which leads to a lobular or interface chronic hepatitis, and results in complications such as cirrhosis, liver failure and hepatocellular carcinoma. Thus, Treg cells, which attenuate inflammation by suppressing the proliferation of effector T cells and the secretion of cytokines, play a fundamental role of controlling hepatitis [54].

In summary, the results of the present study demonstrate that the course of chronic liver disease may not only be modulated by viral components, as has already been described in the literature, but also be regulated by the genes under study, decreasing or inhibiting the regeneration and proliferation of hepatocytes with an essential involvement of the Fas/FasL system and Foxp3^+^ Tregs. These results suggest that regardless of the cause, the course of chronic liver disease may be modulated by the analyzed genes and correlated with an increase in regulatory T cells during the liver damage followed by hepatocyte destruction by Fas/FasL system and subsequent non specific lymphocytic infiltrate accumulation.
